# A New Method of Measuring the Volumetric Change of Alveolar Bone Around Dental Implants Using Computed Tomography

**DOI:** 10.3390/jcm9041238

**Published:** 2020-04-24

**Authors:** Young-Wook Lim, Young-Jun Lim, Bongju Kim, Seung-Pyo Lee

**Affiliations:** 1 Department of Dentistry, School of Dentistry, Seoul National University, Seoul 03080, Korea; ywlim0511@snu.ac.kr; 2 Department of Prosthodontics and Dental Research Institute, School of Dentistry, Seoul National University, Seoul 03080, Korea; 3 Clinical Translational Research Center for Dental Science, Seoul National University Dental Hospital, Seoul 03080, Korea; bjkim016@gmail.com; 4 Department of Oral Anatomy and Dental Research Institute, Seoul National University, Seoul 03080, Korea; orana9@snu.ac.kr

**Keywords:** dental implant, computed tomography, alveolar bone, volumetric change, bone resorption

## Abstract

This study proposes a method for measuring the volumetric change of alveolar bone after dental implant surgery using computed tomography (CT). A total of 40 implants in 20 patients (15 males and 5 females) were selected. The types of implants used were group 1: 24 CMI IS-II Active implants (Neobiotech Co., Seoul, Republic of Korea) and group 2: 16 SLActive Bone Level implants (Institut Straumann AG, Basel, Switzerland). The OnDemand3D software (CyberMed, Seoul, Korea) was used for analysis. The volumetric change of the alveolar bone around an implant fixture is measured as follows: (1) Establish two cylinders: the main cylinder with the implant axis as the central axis (radius of implant + 3 mm) and the error correction cylinder (radius of implant + 1 mm). (2) The height of the cylinder extended from the top of the fixture to a 3 mm coronal portion. (3) Calculate the volumetric change of the alveolar bone (Vd) by subtracting the volume of the error correction cylinder from the main cylinder between CT images taken immediately after the implant placement and 12 months later. After a one-year installation, the volumetric change of alveolar bone, ΔV (cc) had increased in both groups (group 1: −0.011 ± 0.015 cc, group 2: −0.012 ± 0.017 cc) with statistical significance (*p* < 0.05), and the difference between the groups was not statistically significant (*p* > 0.05). This three-dimensional assessment method would be a useful clinical reference for the assessment of marginal bone change after implant surgery.

## 1. Introduction

The marginal bone resorption around the implant fixture caused by periimplantitis exposes a number of problems in many respects [1,2]. In the short term, the peri-implant pocket around the fixture deepens, causing food to be trapped, inflammation, and, in the long term, the bone to be severely absorbed, and the implant to mobilize, resulting in the explantation of the implant.

Adell (1983) [3] proposed that the success of implant therapy should be determined after one year of service because most bone loss occurs during the first 12 months following an abutment connection. The same author reported in a different study that alveolar bone loss during the first year averaged 1.2 mm [4]. 

Since bone level maintenance is one of the most important factors in implant success, alveolar bone loss around implant fixtures has been evaluated based on radiological examinations [5,6,7,8,9]. There were many researchers who studied marginal implant bone loss after dental implant installation in periapical radiographs. It differs from 0.1 to 0.25 mm. Behneke et al. [7] reported that mean annual bone loss a year after installation was 0.1mm. According to Levy et al. [8], it was 0.17 mm, and it was 0.25 mm in Becker’s study [9].

Radiographic examination is useful for assessment of the success of dental implants. However, conventional two-dimensional radiographs such as panoramic and periapical radiographs have limitations in evaluating the success criteria for dental implants. The radiographs can neither evaluate bone change in the buccolingual direction nor accurately show mesiodistal bone resorption [10,11]. 

Computed tomography (CT) can be used as an efficient diagnostic tool for dental implantation. It can evaluate bone quality and adjacent anatomic structures such as the maxillary sinus and inferior alveolar nerve with various advantages over conventional radiographs. First, it is possible to extract the targeted anatomic layer by eliminating the superimposed structures. Second, the high contrast resolution of CT can distinguish differences in physical density of less than 1%, whereas a conventional radiographic image requires approximately a 10% difference in physical density [12]. Third, the multiplanar reformatted imaging technique combines images to generate views in any given plane. Therefore, with CT, the patterns of bone resorption and radiolucency around an implant can be more accurately observed in all directions [10,13].

There have been many studies on bone loss using digital CT in periodontal defect [13,14,15], but few studies have performed a three-dimensional volumetric analysis of peri-implant bone loss using the properties of digital CT imaging in a clinical setting [16,17].

CT, which is widely used in the treatment of dental implants, is primarily used to assess preoperative implantation sites, but not to monitor and evaluate periimplantitis. If it is possible to assess the volumetric change of peri-implant bone using the image characteristics of digital CT, it would be a useful clinical reference for assessing marginal bone resoprion during the maintenance phase.

In this study, a method for measuring the volumetric change of alveolar bone after dental implant placement using CT is proposed to investigate change in peri-implant bone volume in the posterior maxilla during the 12-month, postoperative period.

## 2. Materials and Methods

### 2.1. Data Selection

Twenty partially edentulous patients, 50 to 75 years old, received two implants in the posterior maxillary region from July 2012 to March 2014, at Seoul National University Dental Hospital. Therefore, a final total of 40 implants in 20 patients (15 males and 5 females) were selected for volumetric analysis in this study. The distribution of the implants in the posterior maxilla consisted of 5 first premolars, 21 first molars, and 14 second molars.

The following inclusion/exclusion criteria were applied: ASA type I patients who lost two consecutive posterior maxillary teeth, and periodontally healthy patients 18 years or older who had sufficient alveolar bone volume in the surgical site with a residual bone height of 6 mm or more.

The types of implants used were group 1: 24 CMI IS-II Active implants (Neobiotech Co., Seoul, Korea) and group 2: 16 SLActive Bone Level implants (Institut Straumann AG, Basel, Switzerland). The length and diameter of the implants used in this study were limited to those having 10 mm and 4–5 mm, respectively. Two assigned implants were placed consecutively in the maxillary molar region according to the manufacturer’s instructions in each patient. This means that CMI IS-II Active implants were installed in 12 patients, and SLActive Bone Level implants were installed in 8 patients. If necessary, a sinus augmentation procedure was combined, using a sinus crestal approach kit (SCA kit; Neobiotech Co., Seoul, Korea) with synthetic bone graft materials at the time of placement.

At 4 weeks after implant surgery, the fixtures that showed implant stability quotient (ISQ) values of 65 or more were functionally loaded with a two-unit provisional prosthesis, which was replaced by a monolithic zirconia splinted prosthesis at 2 weeks and 6 months.

Radiographic CT data were collected retrospectively from 40 implants in 20 partially edentulous patients who had CTs immediately after dental implant installation, and 1 year after installation. The study protocol was reviewed and approved by the Institutional Review Board of Seoul National University Dental Hospital (IRB No. CDE12001). 

### 2.2. Methodology

To analyze volumetric change in alveolar bone, CT scans (Somatom Sensation 10, Siemens AG, Forchheim, Germany) were obtained immediately after placement and 12 months after implant insertion, with the following parameters: slice thickness of 1 mm; T1, 0.75 s; 120 kV; and 100 mA/s.

The OnDemand3DTM software (CyberMed, Seoul, Korea) was used for analysis of volumetric change in alveolar bone before and after implant placement. 

The detailed data processing methods are described as follows.

**Figure 1:**
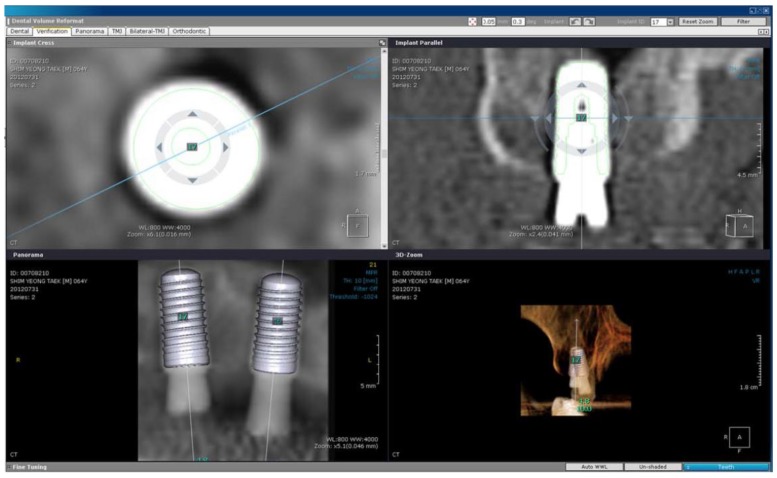
Axial, cross-sectional, panoramic, and 3-dimensional views after positioning of the implant model.

**Figure 2:**
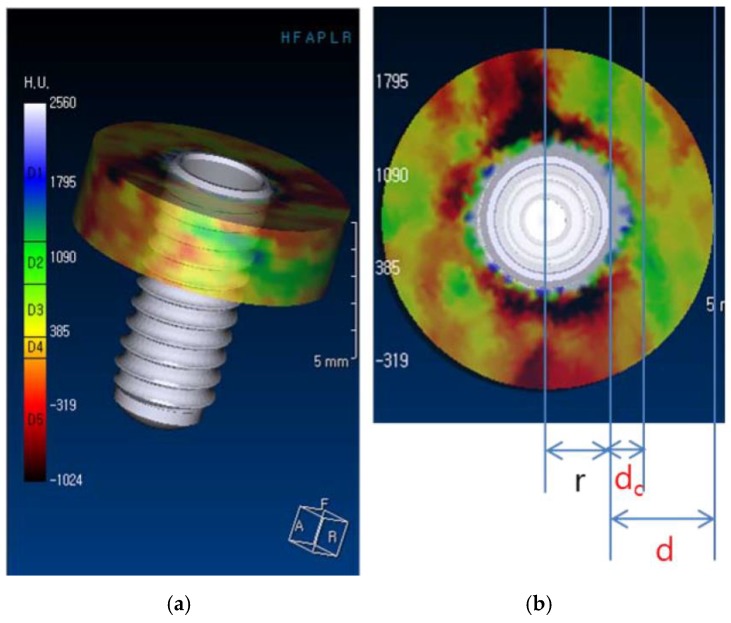
Main and error correction cylinders: (**a**) main cylinder (height = 3 mm) and (**b**) cross-sectional views of the main cylinder and error correction cylinder (r = implant radius, d = 3 mm, and dc = 1 mm).

**Figure 3:**
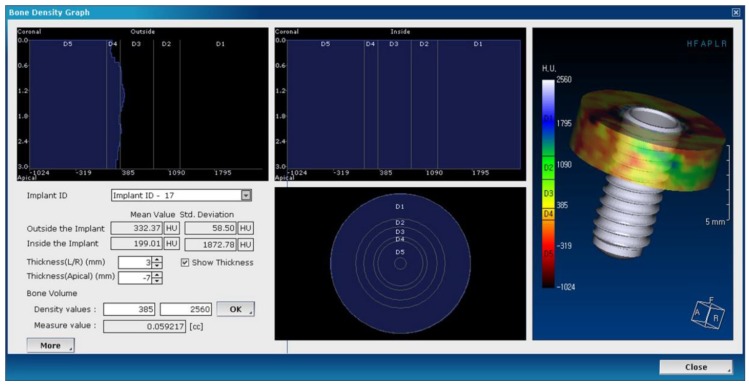
Bone density graph and control panel in the OnDemand3D (Cybermed) software: the values of d and dc can be controlled in the “thickness(L/R)” tab, h in the “thickness(Apical)” tab, and the range of Hounsfield Unit (HU) values in the “density value” tab.

(1) Import the CT file into the OnDemand3D program, and obtain a panoramic view through the center of the placed implant, according to the dental arch shape.

(2) In the acquired panoramic view, import the same implant model in the library folder and superimpose it.

(3) Adjust the fine position of the implant model at the verification tab by setting the rotational angle and position of the model in 0.1 degree and 0.05 mm increments, respectively (Figure 1).

(4) In the program, an axial view, a cross-sectional view, and a 3-dimensional view of placed implants are automatically generated.

(5) Establish two cylinders with the axis of the implant as the central axis, defined as the main cylinder and the error correction cylinder.

(6) Establish a main cylinder whose radius is r + d, where r = radius of implant and d = 3 mm, and set the height of the main cylinder to h = 3 mm (Figure 2).

(7) Establish an error correction cylinder whose radius is r + dc, where r = radius of implant and dc = 1 mm, to reduce errors introduced by radiolucencies around the radiopaque implant fixture in the CT images (Figure 2). The height of the error correction cylinder is set to the same value as that of the main cylinder.

(8) Calculate the volumetric change of the alveolar bone, ΔV, by subtracting the volume of the error correction cylinder from the volume of the main cylinder between the CT images taken immediately after the implant placement and the CT images taken 12 months after implant placement (Figure 3). The bone volume obtained by subtracting the error correction cylinder with the main cylinder is represented by counting the number of voxels of the Hounsfield Unit (HU) of the bone.

### 2.3. Statistical Analysis

A normality test was performed by the Shapiro–Wilk method. A two-tailed student’s *t*-test was used for the volume change of the alveolar bone (paired) or comparison between groups (unpaired). The *p*-value < 0.05 was considered to be significant (SigmaPlot 14.0TM, Systat Software Inc., San Jose, CA, USA).

## 3. Results

**Figure 4:**
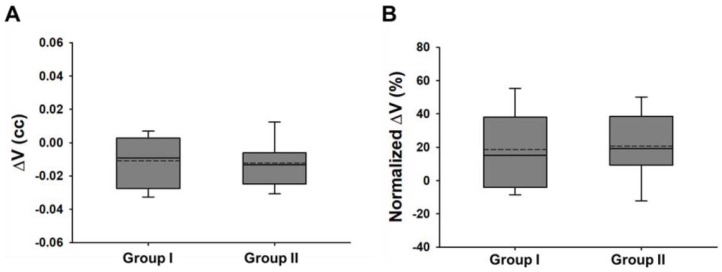
Measurement results of volumetric change of alveolar bone, ΔV (**A**) and normalized ΔV (**B**), after implant installation for 40 implants when height, h, was set to 3 mm.

Measurement results of volumetric change of the alveolar bone (ΔV, normalized ΔV) are summarized in Figure 4 and Table 1.

Volumetric change is the difference between the volumes measured immediately after installation and a one-year installation. The amount of change was measured based on the bone volume immediately after the implant installation. The result is a value that is automatically generated when the determined value on the software is input, and it was confirmed that the same value was obtained by repeating the same process three times.

After a one-year installation, the volumetric change of alveolar bone, ΔV (cc) was increased in both groups (group 1: −0.011 ± 0.015 cc, group 2: −0.012 ± 0.017 cc) with statistical significance (*p* < 0.05). The negative value indicates a volumetric increase of the peri-implant bone.

In the marginal bone region of each group, there was a statistically significant increase rate of 18.7 ± 27.4 and 24.7 ± 22.7%, respectively, compared to immediately after installation, and the difference between the groups was not statistically significant (*p > 0.05*).

Analysis between the two groups for alveolar bone volumetric change showed that SLActive Bone Level implants showed a small amount of more peri-implant bone increase compared to CMI IS-II Active implants, but the difference between the two groups was not statistically significant (*p > 0.05*). 

## 4. Discussion

Based on the results of this study, the volume of alveolar bone around an implant statistically increased 12 months after dental implant placement. This appears to be contradictory to previous studies that reported 0.1–0.25 mm of peri-implant marginal bone loss using periapical radiographs [7,8,9].

This may be due to the size difference of a bony defect caused by drilling or placement of the implant during surgery, which could have affected postoperative bone volume. In this experiment, the maxillary posterior region was composed of cancellous bone with weak bone quality, so that the implants were placed without osteotomy around the upper platform of the implant using a countersink bur.

Most authors recommend that implants installed in the edentulous maxilla of poor-quality bone should be splinted into a rigid one-piece superstructure without cantilevers [18,19,20]. In our study, the splinted prosthesis was mounted on two consecutively implanted fixtures in the maxillary molar region. This would have contributed to reducing the peri-implant bone stress by making the stress distribution favorable for the nonaxial loads applied to the implant.

Another possible reason for the observed differences is angular deviation between the two implant images immediately after surgery and 12 months after insertion. Moreover, additional errors could have been introduced when the implant model was manually superimposed in the three-dimensional CT image. These errors could be reduced with three-dimensional CT image registration. Yi et al. [21] suggested that it is essential to properly align the two images to be evaluated to minimize observer dependency. Mattes et al. [22] proposed an algorithm for three-dimensional positron emission tomography transmission-to-CT registration in the chest, using mutual information as a similarity criterion. After image registration, the volumetric change of alveolar bone can be observed through digital subtraction radiography (DSR), a useful method for assessing small differences on serially obtained radiographs [23].

There are four controllable variables in this proposed method. The distance between the outer surfaces of the implant and the main cylinder, “d,” is related to the region of interest of the volumetric change of the bone. If “d” is set too long, the focus area of the study will be too wide, including the surrounding bone unaffected by surgery. However, if “d’” is too short, the area may be too small to detect the overall change in the alveolar bone. Therefore, in this study, “d” was set to 3 mm so as to not be influenced by other anatomical structures.

Another variable is “dc,” the distance between the outer surfaces of the implant and the error correction cylinder, which should be less than “d”. This variable represents the size of the error correction cylinder. A major reason to introduce the error correction cylinder is a radiolucent rim observed around the implant images, generally formed by bone defects due to drilling or implant placement. As a result, the bone volume measured immediately after surgery is decreased, introducing considerable errors to the results measured. Therefore, it is necessary to set “dc” at a level large enough to reduce a major error factor, but small enough to detect the peri-implant volumetric change of the bone. In this study, because the average width of radiolucencies around the implants was 1 mm, the value of “dc” was set to 1 mm.

Lastly, if the value of “d” sets the horizontal range of the focus area of the study, the height of the cylinder, “h” sets the vertical range of the focus area. Like many other studies in implant dentistry, our focus was on marginal bone change in the crestal region around the implants. Therefore, the value of “h” was set to 3 mm to observe the volumetric change of the crestal region only. 

In short, with the proper setting of variables and control of errors, the proposed method can be a good index of the general change of alveolar bone around implants.

The clinical significance of this study is that the marginal bone loss assessment in all implant retrospective studies currently focuses only on the bone loss of the mesiodistal direction using a two-dimensional periapical radiograph. However, this is a method of measuring the amount of bone change in a single section of the mesiodistal direction obtained as a 2D image, so the pattern of bone change occurring around the implant is not reflected at all [2,16,17].

The most previous implant retrospective studies performed with a periapical radiograph show marginal bone resorption at the crestal region of the implant fixture [7,8,9,24,25]. Our results showed the volumetric increase of marginal bone one year after implant installation in both groups. This result clearly shows the limitations of the two-dimensional assessment of evaluating bone resorption patterns with only mesiodistal direction in periapical radiographs.

This study focused on the introduction of a 3D measurement method using the properties of digital CT imaging to investigate the volumetric change of marginal bone resorption around the implant. All the selected patients had a residual bone height of 6 mm or more, and our study focused on only the marginal bone resorption around the coronal part of the implant fixture. Therefore, the volumetric change around the implant apex was not observed; only the change up to 3 mm from the implant fixture platform towards the apex was investigated.

Volumetric changes of alveolar bone after implant installation in CT is not bone loss in a specific direction just like a buccolingual or mesiodistal direction, but it is a reflection of bone loss in every direction. It can show not only crestal bone loss, but also alveolar bone change, including horizontal bone loss. If the apex of the implants was the focus of interest, alveolar bone change around the apex of the implants also can be observed. Therefore, it could be a good index reflecting the general change of alveolar bone loss around implants.

## 5. Conclusions

Measuring the three-dimensional bone resorption pattern around the implant provides important information for clinicians who maintain the implant-supported prosthesis. 

It is significant that our study suggests a method for evaluating three-dimensional volumetric change of the peri-implant bone using the properties of digital CT. This method would be a useful clinical reference for assessment of marginal bone change after implant surgery.

In the future, as many parts of dental diagnosis and treatment are changed from analog to digital, and the resolution of a digital CT image is improved at the same time, the types and methods of diagnosis using digital images will develop further and become an important tool for diagnosis.

## Figures and Tables

**Table 1:** Change of marginal bone loss after 1 year.

	Group I (Neobiotech)	Group 2 (Straumann)	*p*-Value *	Normality **
Participant number	24	16		
ΔV (cc)	−0.011 ± 0.015	−0.012 ± 0.017	0.798	0.069
Normalized ΔV(%)	18.7 ± 27.4	24.7 ± 22.7	0.808	0.138

* The *p*-values were calculated using the two-tailed *t*-test. ** Normality test was passed (Shapiro–Wilk, *p* > 0.05).
